# Supplementary material for: A computational toolbox for the assembly yield of complex and heterogeneous structures
Source: Nat Commun. 2023 Dec 14;14:8328. doi: 10.1038/s41467-023-43168-4 (PMC10721878; doi:10.1038/s41467-023-43168-4)
Supplement: Supplementary file 1 — Supplementary Information [file 41467_2023_43168_MOESM1_ESM.pdf]

Supplementary Information for "A computational toolbox for the assembly yield of complex and heterogeneous structures"

Agnese I. Curatolo[1,*] Ofer Kimchi[2], Carl P. Goodrich[3], Ryan K. Krueger[1], and Michael P. Brenner[1,4]
[1]*School of Engineering and Applied Sciences, Harvard University, Cambridge, MA 02138*
[2]*Lewis-Sigler Institute, Princeton University, Princeton, NJ 08544*
[3]*Institute of Science and Technology Austria, A-3400 Klosterneuburg, Austria*
[4]*Department of Physics, Harvard University, Cambridge, MA 02138*

* acuratolo@seas.harvard.edu

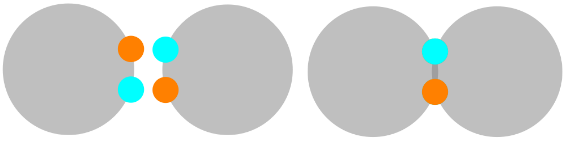

FIG. 1. **Simple 2D model for self-assembly of building blocks with rotational degrees of freedom.** The two identical monomers form a dimer when the orange and cyan patches overlap.

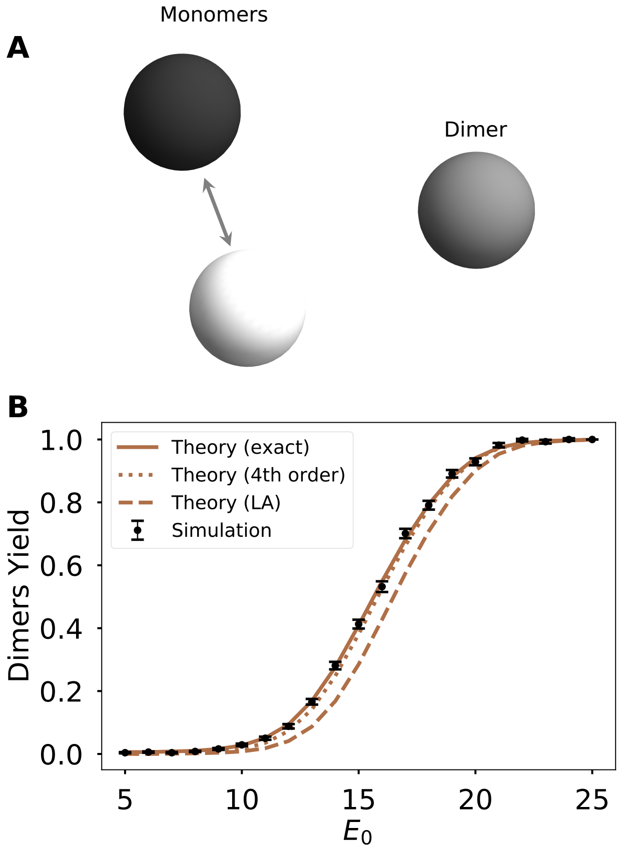

FIG. 2. **Simple model for self-assembly of two spheres in a spherical dimer.** **A**: The black and white monomers are simple spheres that attract each other until they overlap to form a spherical dimer. **B**: Comparison between exact theory, Laplace's approximation (LA), extension to the 4th order and simulation yield. A total of 18 building blocks ($N_1 = N_2 = 9$) were considered in a volume $V = 18\,000\,d^3$ where $d = 1$ is the sphere diameter. Error bars show standard error across 100 replicates. All simulations were performed in the HOOMD-blue simulation package [**?**]. The theoretical yield shown is computed in the canonical ensemble (see SI).

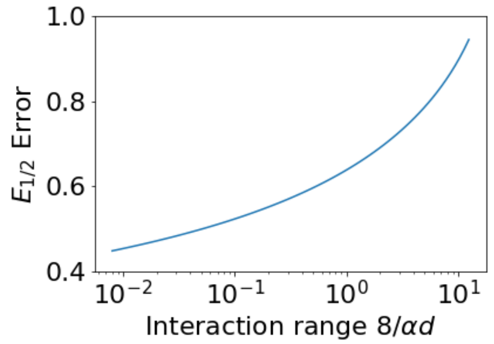

FIG. 3. **Scaling of error with interaction range.** The error due to taking Laplace's approximation to 2nd order can be quantified by measuring $E_{1/2}$, the value of E_0 leading to a dimer yield of 1/2. Here we show this error for different normalized interaction ranges $8/\alpha d$. We consider the same system conditions as in Fig. 2 except here we consider a volume two orders of magnitude larger. The error is relatively small ($\sim k_BT/2$) for short interaction ranges but grows rapidly for interaction ranges greater than the sphere diameter.

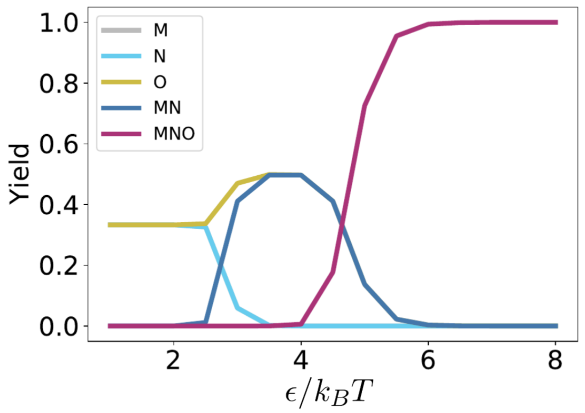

FIG. 4. **Individual cluster yields for the TRAP complex.** We show the yields of the different structures that can form for the system shown in Fig. **??**A as a function of ϵ, when the input concentrations are $c_M^{\text{tot}} = c_N^{\text{tot}} = c_O^{\text{tot}} = 10^{-5}d^{-3}$. The yields of the M and N monomers are almost identical; the former is obscured by the latter in the figure. The yields of the MO and NO dimers are approximately zero for all ϵ and are not shown. At weak interaction energies (low ϵ), the three dimers all form with yield 1/3. At intermediate energies, the MN dimer and O monomer each have yield 1/2. At strong energies (high ϵ), the MNO trimer forms with yield 1.

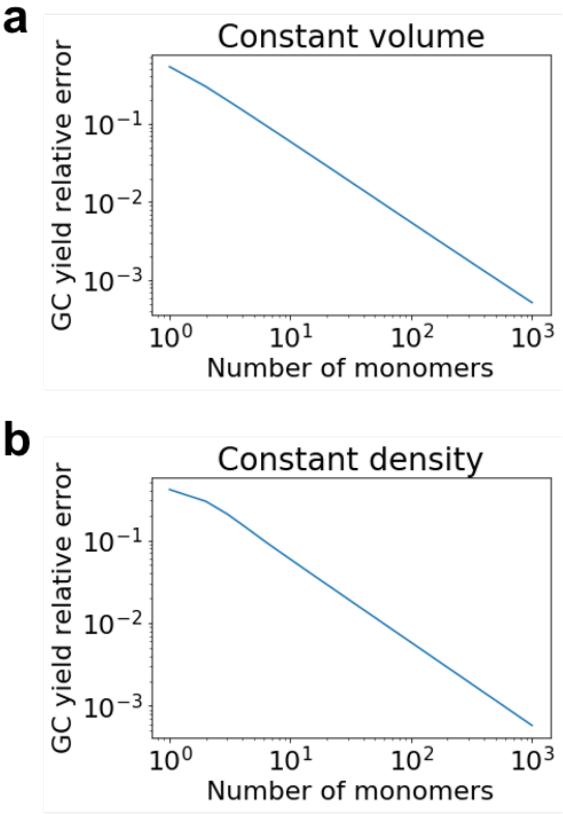

FIG. 5. **Grand canonical ensemble error for finite system sizes.** We consider the same simple dimer system as in Fig. 2A and compare the yields predicted by the canonical and grand canonical (GC) ensembles for different finite system sizes. We place N_m monomers of each kind, with an attractive potential $E_0 = 16$ (in units of k_BT) and with an interaction range of $8/\alpha = 8d/5$. We vary N_m (x-axis) and consider two cases for the volume of the system. **A:** Volume is kept constant at $18,000d^3$ for different values of N_m. **B:** building block density is kept constant at $10^{-3}d^{-3}$. We plot the relative error in the grand canonical ensemble estimate of the yield of the dimers, defined as $(Y_C - Y_{GC})/Y_C$ where Y_{GC} is the yield as predicted by the grand canonical ensemble, and Y_C is the yield predicted by the canonical ensemble. We find that this error decreases as a power law as the number of building blocks increases.
